# Clinical diagnosis of TB: further examples of under- and over-diagnosis

**DOI:** 10.5588/pha.25.0023

**Published:** 2025-09-03

**Authors:** A.D. Harries

**Affiliations:** ^1^ Centre for Operational Research, International Union Against Tuberculosis and Lung Disease, Paris, France;; ^2^ Department of Clinical Research, Faculty of Infectious Diseases and Tropical Medicine, London School of Hygiene and Tropical Medicine, London, UK.

**Keywords:** tuberculosis, smear-negative pulmonary TB, extrapulmonary TB, Malawi, operational research, HIV-associated tuberculosis, LAM

Dear Editor,

I thank Singini et al. for their informative article on misdiagnosis and overdiagnosis of clinically diagnosed TB.^1^ Twenty five years ago, within the Malawi National TB Programme, we found similar issues. At that time, clinically diagnosed TB was subdivided into smear-negative pulmonary TB (PTB) and extrapulmonary TB (EPTB). In 2000, we visited all 43 government and mission hospitals in Malawi involved in the management of TB, and identified adults aged ≥15 years registered and on treatment for smear-negative PTB or EPTB. We assessed through case notes, TB treatment cards and TB registers whether national guidelines for diagnosing these two diseases had been followed. We took a clinical history, performed a clinical examination and re-examined/reclassified chest radiographs or other radiographs as normal, abnormal but not consistent with TB, and abnormal and consistent with TB, as done by Singini and colleagues.^1^ We decided whether the diagnosis of TB was correct or not, and if not, another diagnosis was made. If the diagnosis of TB was clearly incorrect, anti-TB treatment was stopped and appropriate treatment commenced. If there was any doubt, anti-TB treatment was continued.

For adults with smear-negative PTB, over 90% had been assessed according to national guidelines and had appropriate clinical features, had undergone sputum smear examination and chest radiography, and received a course of antibiotics.^2^ In 86% of patients the diagnosis of TB was correct: 78% had smear-negative PTB and 8% had EPTB (mainly pleural effusion and pericardial effusion). However, in 14% the diagnosis was not TB, the main alternative diseases being pneumonia, AIDS or left ventricular failure. For adults with EPTB, only 57% had all the appropriate procedures and investigations for that particular type of EPTB.^3^ The diagnosis of TB was correct in 83% of patients. However, in 16% of patients, an alternative non-TB diagnosis was made (in 1% it was not possible to arrive at a decision). For non-TB diagnoses, the three common mistakes were: diagnosing heart failure as TB pleural effusion or TB pericardial effusion, diagnosing HIV-related persistent generalised lymphadenopathy as TB lymphadenopathy, and diagnosing chronic liver disease as TB ascites. The mistakes were mainly due to poor clinical assessment and incorrect radiographic interpretation, the latter also identified as a major challenge by Singini et al.^1^ In low-resource settings, where expert radiographic advice is in short supply, computer-aided diagnosis (CAD) systems could potentially improve the situation.

There is also, however, another side to the coin! A systematic review and meta-analysis of post-mortem studies in Africa and Asia found that approximately 40% of adult HIV/AIDS-related deaths were due to TB, largely disseminated disease.^4^ In nearly half of these cases, TB was undiagnosed until after death. Thus, not only do we over-diagnose TB but we also under-diagnose TB, especially in high HIV-burden areas. Disseminated TB in HIV-infected persons is a life-threatening condition. The use of urine Xpert and urine lipoarabinomannan (LAM) is useful in diagnosing disseminated TB in HIV-infected hospitalised in-patients,^5^ and should be more widely used in high HIV/TB-burden areas.
